# Draft Genome Sequences of Three Pseudomonas fluorescens Strains Isolated from Marine Sponges Harvested off the West Coast of Ireland

**DOI:** 10.1128/MRA.00457-20

**Published:** 2020-05-21

**Authors:** José A. Gutiérrez-Barranquero, María L. Parages, Alan D. W. Dobson, F. Jerry Reen, Fergal O’Gara

**Affiliations:** a BIOMERIT Research Centre, School of Microbiology, University College Cork, Cork, Ireland; b School of Microbiology, University College Cork, Cork, Ireland; c Environmental Research Institute, University College Cork, Cork, Ireland; d Telethon Kids Institute, Perth Children’s Hospital, Perth, WA, Australia; e School of Pharmacy and Biomedical Sciences, Curtin Health Innovation Research Institute, Curtin University, Perth, WA, Australia; Georgia Institute of Technology

## Abstract

Three *Pseudomonas* sp. strains isolated from marine sponges have shown potential quorum sensing inhibition (QSI) activity. We sequenced the draft genomes of the three strains with the goal of determining which genes or gene cluster(s) could be potentially involved in the QSI activity. Average nucleotide identity (ANI) and phylogenetic analysis classified the three strains as belonging to the Pseudomonas fluorescens species.

## ANNOUNCEMENT

*Pseudomonas* is a Gram-negative bacterial genus belonging to the *Gammaproteobacteria* class, whose members are known to colonize and survive in a wide range of diverse environments, mainly due to inherent broad metabolic diversity (1, 2). This bacterial genus encompasses different species involved in the degradation of xenobiotic compounds (3), human and plant pathogenesis (4, 5), and plant growth promotion and biocontrol (6). Recently, the role of some *Pseudomonas* sp. strains in the inhibition of quorum sensing (QS) signaling systems has been reported (7–9). Three *Pseudomonas* sp. strains (B98C39, B98SK52, and B98SM8) were isolated from 2 different marine sponges, belonging to the *Hexactinellida* class, that were collected off the west coast of Ireland as part of a marine biodiscovery cruise in May 2010. The isolation of these *Pseudomonas* sp. strains and their QS inhibition (QSI) activities were described previously (9). However, the genetic basis of the QSI activities remained unknown. Therefore, in order to identify which genes or gene cluster(s) could potentially be involved in the QSI activity, the draft genome sequencing of these strains was completed.

Overnight shaking cultures grown in lysogeny broth at 23°C for the three *Pseudomonas* sp. strains were used to perform total DNA isolation using the UltraClean microbial DNA isolation kit (MO BIO Laboratories, Inc., Carlsbad, CA, USA). DNA libraries were prepared using a TruSeq exome library preparation kit. The draft genome sequencing was performed by the Beijing Genomics Institute (China) using the Illumina HiSeq 2000 sequencing platform with paired-end reads and a read length of 90 bp for B98C39. The HiSeq 4000 platform, with paired-end reads and a read length of 150 bp, was used for B98SK52 and B98SM8. In order to obtain high-quality reads for assembly, the FASTA/Q file manipulation tool readfq.v5 (10, 11) was used for quality trimming using the same parameters as described previously (10). Thus, the high-quality-filtered reads were all 90 bp in the case of B98C39 and 150 bp in the cases of B98SK52 and B98SM8. The assembly of high-quality filtered reads was performed using SOPAdenovo v2.04 with default parameters. Genome sequence annotation and gene identification were carried out with the Rapid Annotations using Subsystems Technology (RAST) v2.0 server (using default parameters and RASTtk for the annotation scheme) (12, 13) and the NCBI Prokaryotic Genome Annotation Pipeline (PGAP) (using default parameters). The main characteristics of the draft genome sequences obtained, including their accession numbers, are summarized in Table 1.

**TABLE 1:** Accession numbers and genome assembly features

*P. fluorescens* strain	GenBank accession no.	Genome size (Mb)	SRA accession no.	No. of contigs	No. of scaffolds	*N*_50_ (bp)	Mean coverage (×)	No. of CDS^*a*^	G+C content (%)
B98C39	JAAXCP000000000	6.21	SRX8046944	282	51	44,154	41.50	5,459	59.9
B98SK52	JAAXCO000000000	6.21	SRX8046945	73	71	259,749	40.90	5,566	59.8
B98SM8	JAAXCN000000000	6.21	SRX8046946	97	97	149,291	42	5,568	59.8

a CDS, coding DNA sequences.

**Fig 1:**
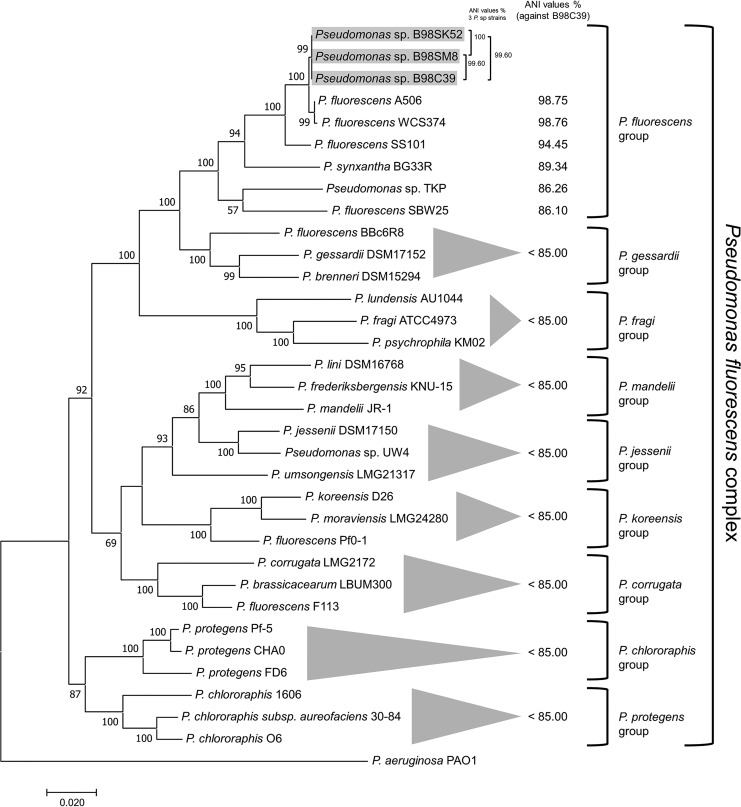
Phylogenetic analysis using the complete nucleotide sequences of the housekeeping genes *gyrB* and *rpoD*. Thirty-three *Pseudomonas* strains belonging to the eight phylogenetic groups within the Pseudomonas fluorescens complex were used. Phylogenetic distribution was determined by the maximum likelihood method and the Tamura-Nei model, with 100 bootstrap replicates, using MEGA7. The three Pseudomonas fluorescens strains sequenced in this study are highlighted with gray boxes. The P. aeruginosa PAO1 strain was used as an outgroup. ANI values are represented as percentages. According to previous work (20), the ANI value threshold for differentiating species in the Pseudomonas fluorescens complex is 85%. Thus, an ANI value over 85% is considered to indicate the same species. The data for the strains used to generate this figure were obtained from the NCBI database, with the following accession numbers: CP003041, CP007638, AHPN00000000, AHPP00000000, CP006852, AM181176, AKXH00000000, UYXZ00000000, VFIL01000000, CP017687, BDAB00000000, CP049044, JYLB01000000, CP023466, CP005960, NIWT01000000, NC_019670, LT629767, CP014947, LT629788, CP000094, LT629798, CP012680, CP003150, CP032358, CP003190, CP031396, CP011110, CM001490, CM001559, and AE004091.

Genome mining of the three strains identified orthologs of the *quiP* (14) and *pvdQ* (15) genes, both of which encode acylase enzymes that were previously shown to control the QS system in Pseudomonas aeruginosa through the degradation of *N*-3-oxododecanoyl-homoserine lactone. Furthermore, a nonribosomal peptide synthetase secondary metabolite gene cluster was identified using antiSMASH bacterial version 5.0 (16); the cluster was related to the production of salicylic acid and pseudomonine metabolites (17). Interestingly, salicylic acid was reported previously to inhibit the production of *N*-acylhomoserine lactones in bacteria (18). Considering these results, further *in vitro* investigations will be required to elucidate which enzymes or metabolites could be involved in the QSI activity of these *Pseudomonas* sp. strains. Phylogenetic analysis and pairwise ANI analysis using JSpeciesWS were performed (19) (Fig. 1). The sequenced strains were classified as belonging to the species Pseudomonas fluorescens.

### Data availability.

The draft genome sequences discussed in this work have been deposited at DDBJ/ENA/GenBank and the Sequence Read Archive (SRA). The corresponding accession numbers are listed in Table 1.
